# Aspirin in venous leg ulcer study (ASPiVLU): study protocol for a randomised controlled trial

**DOI:** 10.1186/s13063-016-1314-4

**Published:** 2016-04-11

**Authors:** Carolina D. Weller, Anna Barker, Ian Darby, Terrence Haines, Martin Underwood, Stephanie Ward, Pat Aldons, Elizabeth Dapiran, Jason J. Madan, Paula Loveland, Sankar Sinha, Mauro Vicaretti, Rory Wolfe, Michael Woodward, John McNeil

**Affiliations:** Department of Epidemiology and Preventive Medicine, School of Public Health and Preventive Medicine, Monash University, Melbourne, VIC 3004 Australia; School of Medical Sciences, RMIT University, Bundoora, VIC 3083 Australia; Clinical Trials Unit, University of Warwick, Coventry, CV4 7AL UK; Private Practice Clinic, Prince Charles Hospital, Chermside, QLD 4032 Australia; Chronic Wound Clinic, Caulfield Hospital, Caulfield, VIC 3162 Australia; Aged Care Services, Heidelberg Repatriation Hospital, Heidelberg West, VIC 3081 Australia; School of Medicine, University of Tasmania, Hobart, TAS 7000 Australia; Westmead Clinical School, Westmead Hospital, Westmead, NSW 2006 Australia; Faculty of Medicine, Nursing and Health Sciences, School of Public Health & Preventive Medicine, The Alfred Centre, 99 Commercial Road, Melbourne, 3004 Australia

**Keywords:** Aspirin, Venous leg ulcers, Compression, Healing

## Abstract

**Background:**

Venous leg ulceration is a common and costly problem that is expected to worsen as the population ages. Current treatment is compression therapy; however, up to 50 % of ulcers remain unhealed after 2 years, and ulcer recurrence is common. New treatments are needed to address those wounds that are more challenging to heal. Targeting the inflammatory processes present in venous ulcers is a possible strategy. Limited evidence suggests that a daily dose of aspirin may be an effective adjunct to aid ulcer healing and reduce recurrence. The Aspirin in Venous Leg Ulcer study (ASPiVLU) will investigate whether 300-mg oral doses of aspirin improve time to healing.

**Methods/design:**

This randomised, double-blinded, multicentre, placebo-controlled, clinical trial will recruit participants with venous leg ulcers from community settings and hospital outpatient wound clinics across Australia. Two hundred sixty-eight participants with venous leg ulcers will be randomised to receive either aspirin or placebo, in addition to compression therapy, for 24 weeks. The primary outcome is time to healing within 12 weeks. Secondary outcomes are ulcer recurrence, wound pain, quality of life and wellbeing, adherence to study medication, adherence to compression therapy, serum inflammatory markers, hospitalisations, and adverse events at 24 weeks.

**Discussion:**

The ASPiVLU trial will investigate the efficacy and safety of aspirin as an adjunct to compression therapy to treat venous leg ulcers. Study completion is anticipated to occur in December 2018.

**Trial registration:**

Australian New Zealand Clinical Trials Registry, ACTRN12614000293662

## Background

Venous leg ulcers (VLUs) are a common and costly problem, usually managed in general practice and community settings with variability in clinical practice [1]. Age-related venous leg ulceration is the most common cause of lower limb ulceration in developed countries, with an overall prevalence between 1.65 and 1.74 % and substantially higher rates in adults of age 65 and older [2]. These ulcers become chronic due to the underlying pathophysiology and are associated with pain and heavy exudate [3]. In 2010, an estimated 400,000 Australians were treated for VLUs, translating into costs of more than AU$3 billion per year [4]. People with chronic venous insufficiency (CVI) are prone to developing VLUs on the lower legs, which can occur spontaneously or after minor trauma [5]. Up to 70 % of VLUs will heal within 12 weeks if adequate compression is applied and patients adhere to compression treatment [6]. People with VLUs also often suffer from diabetes and obesity, further impacting the healing [7]. The natural history of ulceration is a cycle of healing and recurrence, which has considerable impact on an individual’s health, quality of life, and socioeconomic costs [8, 9]. Increasing life expectancy means that more people will be living with VLUs in the future, increasing the financial and healthcare burden of this already costly chronic disease [10, 11].

### Current treatment of venous leg ulcers

Best practice VLU treatment is a firm graduated compression bandage to aid venous return [12]. This assists by reducing venous hypertension, enhancing venous return, and reducing peripheral oedema. Data shows that VLUs heal more quickly with compression than without [12]. Treatment inconsistencies regarding the bandage application and limited patient adherence to compression therapy have been identified as limiting factors to healing [1]. Many healed ulcers recur within 3 months of healing, possibly due to a prolonged inflammatory response [6]. Aspirin is a widely used and relatively well-tolerated drug. It has the potential to reduce inflammation via generalised COX inhibition.

Classical signs of inflammation have been observed in biopsies and plasma samples in experimental models of venous disease [13], and evidence supports the hypothesis that, in humans, chronic venous disease is associated with the inflammatory cascade [14]. The cascade begins with increased vascular permeability and progresses to the adhesion of leukocytes and platelets to the endothelium. Over time, increased cell apoptosis, degradation of the basement membrane and extracellular matrix proteins, and vascular restructuring of the venous varicosities occurs [15]. High-level evidence shows larger wounds of longer duration (>10 cm^2^ and > 12 months old) at the first visit have only a 20 % chance of healing by the 24th week of care, whereas a wound that is smaller than 10 cm^2^ and has been present for less than 12 months has a better (70 %) chance of healing by 24 weeks [10, 16]. A perpetual inflammatory state may contribute to difficult-to-heal chronic wounds [17]. VLUs may achieve better healing and be less likely to occur due to anti-inflammatory effects if the compression bandage is used concomitantly with aspirin [18].

### Aspirin as a novel treatment in VLUs

Aspirin has several actions potentially capable of influencing the progression of VLUs through the suppression of inflammation. It inhibits the enzyme cyclooxygenase (COX), thereby blocking the synthesis of several potent stimulators of inflammation [19]. In vivo studies suggest that 300 mg aspirin suppresses inflammatory markers and may promote ulcer healing [20]. Aspirin also reduces prostaglandin-2 and thromboxane A2, which are involved in platelet aggregation [19]. The production of these compounds underpins part of the anti-inflammatory effect of aspirin, along with the potential to inhibit both leukocyte activation and platelet function [18]. The effect of low-dose aspirin on serum levels of inflammatory cytokines, such as interleukin 6 (IL-6) and tumour necrosis factor-alpha (TNF-a), is not entirely clear at present. In one study of aspirin in people with VLUs, investigators reported increased fibrinogen and an increased coagulation rate, which increased the healing rates [21]. Some publications report little or no effect on the serum levels of pro-inflammatory cytokines, while others show reduced serum IL-6 and TNF-a with 2 weeks treatment using 300 mg/day aspirin [20].

### Previous studies of aspirin in venous leg ulcers

Two small, randomised, controlled trials (RCTs) suggest aspirin may be effective in speeding ulcer healing and may reduce the ulcer recurrence, although the quality of this evidence is low. An RCT conducted in the United Kingdom [21] (*n* = 20) reported that daily oral administration of aspirin (300 mg) with compression decreased the time to ulcer healing (*p* < 0.01) and the proportion of participants healed when compared to placebo with compression bandaging over a 4-month period. Thirty-eight per cent of the aspirin participants reported complete healing compared with 0 % in the placebo group (*p* < 0.007). Improvement, assessed by a reduction in wound size, occurred in 52 % of the aspirin group compared with 26 % with placebo (*p* < 0.007). The potential benefits of aspirin as an adjunct to compression were identified, but the sample size was small, and the mechanism by which aspirin improved healing or the effect on recurrence was not reported [20]. A more recent Spanish RCT [22] (*n* = 51) compared daily administration of aspirin (300 mg) in addition to the use of a compression bandage with compression alone over a 5-month period [22]. Little difference was observed in the complete healing between the groups (21/28 aspirin and 17/23 compression alone); however, the average time to healing was shorter (12 weeks in the aspirin group versus 22 weeks in the compression-only group), and the average time to recurrence was longer in the aspirin group (39 days (SD 6.0) for aspirin versus 16.3 days (SD 7.5) for compression alone). No information regarding the placebo was reported.

The ASPiVLU study in Australia will investigate the effects of aspirin in people with VLUs in a large, randomised, double-blinded, placebo-controlled trial. We have selected a daily dose of 300 mg aspirin, as current evidence was derived from studies using this dose.

### Study objectives

The primary objective is to determine whether daily aspirin (300 mg) as an adjunct to compression improves the time to healing of the target ulcer in a 12-week treatment period.

The secondary objective is to determine the effects of aspirin on ulcer recurrence, wound pain, quality of life and wellbeing, adherence to study medication, adherence to compression therapy, serum inflammatory markers, hospitalisations, and adverse events at 24 weeks.

## Methods/design

### Study design

ASPiVLU is a randomised, double-blinded, multicentre, placebo-controlled, clinical trial to determine the clinical effectiveness of aspirin as an adjunct to compression in healing venous leg ulcers.

### Study setting and participants

The study population consists of adult patients living in Australia. The study is being conducted in four states (Victoria, New South Wales, Queensland, and Tasmania). Potential participants will be identified from community nursing settings, general practice clinics, and hospital outpatient wound clinics. All eligible patients will be identified at six participating ASPiVLU study wound clinics and offered study participation.

### Inclusion criteria

Inclusion criteria are as follows:

- Age 18 years and older
- Have one or more leg ulcers in the presence of venous insufficiency confirmed by clinical assessment and/or duplex ultrasound
- Target ulcer (largest ulcer if more than one) is separated from the other ulcers by at least 1 cm
- Target ulcer present for at least 6 weeks or has prior history of venous ulceration
- Target ulcer area ≥ 1 cm^2^ to ≤ 20 cm^2^ as measured by digital planimetry techniques
- Ankle brachial pressure index (ABPI) measure of ≥ 0.7 mmHg *or* systolic toe pressure ≥ 50 mmHg to exclude arterial insufficiency
- Able to provide informed consent (decision made according to the medical practitioner’s clinical judgement)

### Exclusion criteria

Exclusion criteria include the following:

- Current, daily aspirin use
- Aspirin intolerance
- Any existing condition or treatment that is a contraindication to aspirin therapy or to participate in the trial (decision made according to the medical practitioner’s clinical judgement)
- Concurrent use of any other antiplatelet or anticoagulation therapy
- Pregnancy or breastfeeding

### Consent

Written information, in the form of a patient information and consent form, will be provided after verbal explanation of the research study. Patients will have the opportunity to discuss participation with research staff prior to enrolment. Written informed consent will be obtained from each participant prior to randomisation.

### Screening assessment

After giving consent, the patients will be screened at wound clinics to verify eligibility. Eligibility will be confirmed by the wound clinic doctor. Prior to randomisation, baseline data, including age, gender, smoking status, ethnicity and employment status, medical history and current medication use, will be recorded. A physical examination by a wound clinic consultant will include an assessment of the target ulcer, measurement of the wound surface area, and digital photography of the wound.

### Intervention

Study participants will be allocated to either active treatment with aspirin at a dose of 300 mg daily or inactive placebo. Study tablets are enteric-coated, un-scored, white tablets with identical appearance, provided by Bayer Pharma AG for the ASPiVLU trial. Participants will be advised to take one tablet each morning approximately half an hour before other medications to avoid potential drug interactions. Participants will continue to take the study medication daily for 24 weeks, regardless of whether or not the target ulcer heals.

### Compression therapy

All participants will be treated with compression therapy (the system of choice will be guided by clinician discretion and patient preference) for 12 weeks or until complete healing of the target ulcer occurs. Details of the compression type will be noted in the case report form. Once ulcer healing is achieved, the participants will be fitted with below-knee compression stockings that deliver between 23 mmHg to 32 mmHg compression at the ankle to prevent ulcer recurrence.

### Randomisation

Patients will be randomly assigned to aspirin or placebo via an electronic data capture system (EDC). The randomisation list will be generated by an independent statistician and implemented together with medication number selection by the database programmer. Randomisation codes will be allocated via a password-protected system.

### Allocation concealment and implementation

The study medication will be packaged in identical containers. Each container will be pre-labelled (by an independent drug-packaging group contracted to provide the study medication) with a study identifier according to the randomisation schedule. Allocation will be stratified by wound clinic and wound size as measured by the Margolis index [10]. The Margolis index is a prognostic score for venous ulcer healing derived from dichotomous categorisations of the ulcer area and duration of the current ulcer. Hence, stratification aims to avoid baseline imbalances in the ulcer size and duration, which are highly predictive of outcome. Stratification based on clinic site will avoid variance in any baseline characteristics related to the geographical location or local clinical practice. The study investigators and the research nurses distributing the medication and assessing outcomes will remain blind to the treatment allocation.

### Un-blinding

Un-blinding will occur in the event of a clinical emergency in which the knowledge of the medication taken is essential for the participant’s clinical management. The code can be broken by contacting the Trial Pharmacist via the ASPiVLU Trial Centre. Reasons for un-blinding will be recorded, and participants will be encouraged to resume their assigned medication, if possible, after their immediate condition has resolved.

### Primary outcome measure

The primary outcome is the time to healing of the target ulcer at 12 weeks after randomisation. Healing is defined as 100 % epithelialisation, with no exudate or scab. Proof of healing will be confirmed by an independent expert review of the digital photos of the ulcer. Time to healing will be measured in weeks from the date of randomisation.

The target ulcer is defined as the largest ulcer at baseline, which is separated from other ulcers by at least 1 cm and has a surface area between 1 cm^2^ and 20 cm^2^.

### Secondary outcome measures

The following secondary outcomes will be assessed in all participants at 24 weeks after randomisation.

#### Recurrence of target ulcer

Recurrence is defined as healed at 12 weeks and recurrence free at 24 weeks. After healing, the participants will be followed up to assess target ulcer recurrence. This will occur via monthly phone calls by the research staff from the date of healing to 24 weeks from randomisation.

#### Wound pain score

(Baseline and weekly until the wound is healed or to 12 weeks, whichever comes first, and then monthly via phone call until the study completion at 24 weeks): The participants’ self-reported assessments of pain associated with their VLUs in the past week will be measured with an 11-point (0–10) numerical rating scale, where a score of ‘0’ represents no pain, and ‘10’ represents the maximal pain imaginable.

#### Health-related quality of life and wellbeing index

(Baseline, 12 weeks, and 24 weeks): The EQ-5D is a health-related quality of life and wellbeing instrument. The EQ-5D-5 L is a widely used generic preference-based measure of health outcomes [23]. It consists of a descriptive system together with a visual analogue 0–100 scale. It assesses five dimensions of health – mobility, self-care, usual activities, pain/discomfort, and anxiety/depression. Participants are asked to rate each dimension as one of five levels (no problems, slight problems, moderate problems, severe problems, or extreme problems). This can result in 3125 (5^5^) possible health states, which can be converted to health utilities using nation-specific tariffs, which have been developed for a number of countries [24].

#### Adherence to study medication

(Weekly from baseline until healed or to 12 weeks, whichever comes first, and then monthly to 24 weeks from randomisation): Participants will be asked to self-report trial medication adherence and to present medication containers for pill counts at 24 weeks.

#### Adherence to compression treatment

(Weekly from baseline until healed or to 12 weeks, whichever comes first, and then monthly to 24 weeks from randomisation): Participants will be asked to report whether they adhered to wearing the compression on a categorical scale of ‘wear everyday’, ‘wear ≥ 3 days/week’, ‘wear ≤ 2 days per week’, and ‘do not wear’.

#### Adverse events

(Weekly until healed or to 12 weeks, whichever comes first, and then monthly via phone call until the study completion at 24 weeks): Adverse events will be elicited by open-ended questions and recorded in the adverse events log.

#### Hospitalisation

At each contact, participants will be asked to report hospitalisation that occurs for any reason during the 24-week study period.

#### Serum inflammatory markers

(Baseline, 12 weeks, and 24 weeks): At these time points, 20 ml of blood will be collected from participants. Prior to assay, the blood will be centrifuged as per standard operating procedures and frozen at −80 °C for storage. Assays of inflammatory markers and cytokines will assess interleukin (IL)-1β, IL-2, IL-4, IL-5, IL-6, IL-10, IL-12, IL-13; IFN-γ; TNF-α; and, possibly, MCP1; CRP; and P-selectin (subject to costs).

**Fig. 1:**
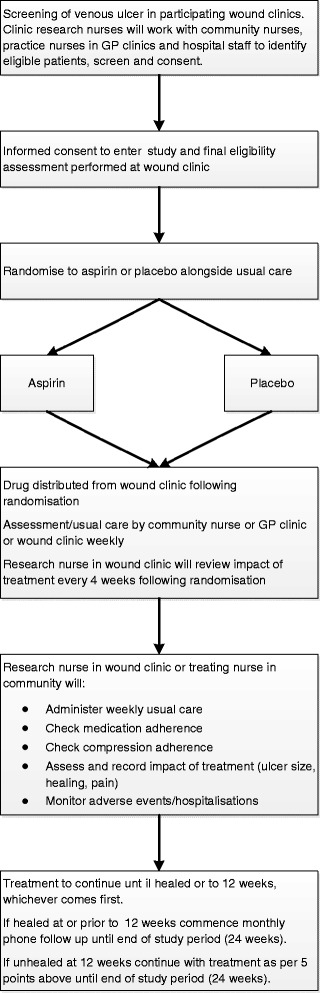
Schematic of the Aspirin in Venous Leg Ulcer trial (ASPiVLU) design

**Fig. 2:**
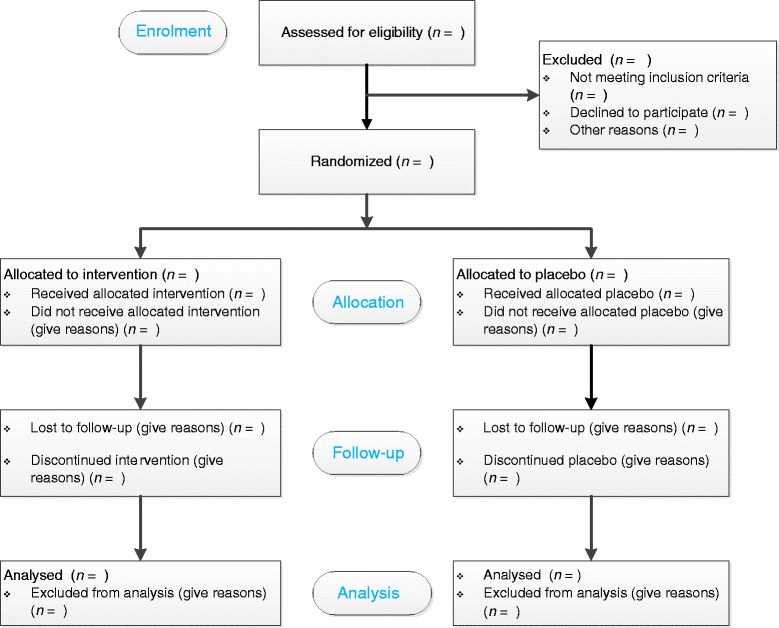
The Aspirin in Venous Leg Ulcer (ASPiVLU) trial flow diagram

A schematic of the ASPiVLU trial design is provided in Fig. 1, and an overview of the study schedule is provided in Table 1. The CONSORT flow diagram is provided in Fig. 2.

**Table 1:** Study schedule

Measurement/activity	Screening^a^	Treatment period in weeks													Follow-up period	
		*(Weekly wound management and assessments until target ulcer healed OR 12 weeks – whichever occurs first. The 12-week visit is mandatory for all participants regardless of ulcer status.)*													*(from week 12 until week 24)*	
		Recruitment & Baseline Assessment (Day 1)	Wk 1	Wk 2^b^	Wk 3^b^	Wk 4^b^	Wk 5^b^	Wk 6^b^	Wk 7^b^	Wk 8^b^	Wk 9^b^	Wk 10^b^	Wk 11^b^	Wk 12	Monthly	Wk 24/end of study
Informed consent		X														
Eligibility criteria	X															
Recruitment & randomisation		X														
Target ulcer assessment – general features		X	X	X	X	X	X	X	X	X	X	X	X	X^3^		
Wound pain score		X	X	X	X	X	X	X	X	X	X	X	X	X^3^	X	X
Target ulcer assessment – size (tracing) & photo		X				X				X				X^3^		
Medical history including demographics & medications		X														
Physical examination including vital signs		X														
QOL assessment (EQ-5D-5L)		X												X		X
Serum sample		X												X		X
Trial medication dispensed		X														
Ulcer care education		X														
Adherence to compression (or hosiery)			X	X	X	X	X	X	X	X	X	X	X	X	X	X
Adherence to trial medication			X	X	X	X	X	X	X	X	X	X	X	X	X	X
Concomitant/new medication			X	X	X	X	X	X	X	X	X	X	X	X	X	X
Adverse events including hospitalisation			X	X	X	X	X	X	X	X	X	X	X	X	X	X
Wound assessment			X	X	X	X	X	X	X	X	X	X	X	X		
Ulcer prevention education			X^2^	X^2^	X^2^	X^2^	X^2^	X^2^	X^2^	X^2^	X^2^	X^2^	X^2^	X		
Target ulcer recurrence assessment															X^2^	X^2^
Refer to wound clinic														X^3^	X^4^	X^4^
Reconciliation of medication																X

^a^Screening to be conducted within 14 days of baseline (day 1) assessment

^b^Assessment conducted only if the target ulcer unhealed

X^2^ = if healed

X^3^ = if not healed

X^4^ = if ulcer has recurred *(this applies to participants whose target ulcer recurs after initial healing is achieved)*

### Participation discontinuation

If a participant withdraws from the study, the reason will be documented, and the participant will be referred for routine care to the wound clinic, if required. Participants will be asked to allow measurement of the target ulcer at routine treatment visits for ascertainment of outcomes. Participants unwilling to continue participation during the follow-up period will be asked for permission to allow investigators to collect information from their medical records.

### Follow-up of participants ‘off protocol’

‘Off protocol’ is defined as those *study participants who cease trial medications on clinical grounds or commence on aspirin therapy for other reasons*. This may be short-term or for the remainder of the study. Regardless of the decision to continue with study medication, these participants will be asked to participate in all scheduled follow-up contacts, as if they were maintaining full participation. Those who are unwilling or unable to do this will be asked to agree to phone call or mail follow-up, and/or permission for study staff to continue surveillance of their clinical records.

### Sample size

We estimate that 268 participants will be required. Based on a previous compression bandaging study, we expect 50 % of ulcers to have healed at 12 weeks in the placebo group [15]. Aspirin is expected to increase this additively by 20 %; that is, we expect 70 % of the aspirin group to be healed at 12 weeks [20, 22]. Twelve-week survival rates of 0.5 and 0.3 in the placebo and aspirin groups, respectively, translate to cumulative hazard rates of 0.7 and 1.2, respectively, and a hazard ratio of 1.74. For 90 % power to detect this hazard ratio in a two-sided (alpha = 0.05) test for time to healing, analysed by a log-rank test, we require 121 people per group. Allowing for 10 % loss to follow-up, we will recruit *n* = 134 participants per group. With this sample size, and the same assumptions about percentage healed, we have 87 % power for a two-sample test of the proportion healed at 12 weeks.

For the secondary outcome of healed at 12 weeks and recurrence free at 24 weeks, we anticipate 60/121 (approximately 50 %) in the placebo group to be healed at 12 weeks and 85/121 (approximately 70 %) in the aspirin group to be healed. Of these participants with healed ulcers, in the placebo group, we expect 24 (40 % of 60) to recur by 24 weeks from baseline, i.e. 36/121 to be healed at 12 weeks and recurrence free at 24 weeks. We have 90 % power to detect a difference between the placebo and aspirin groups if the proportion healed at 12 weeks and recurrence free at 24 weeks in the aspirin group is 51 % (*n* = 62) of 121 participants.

### Data management and statistical analyses

Results from the trial assessments will be recorded in the electronic case report forms (CRF) via an electronic data capture (EDC) system. The REDCap EDC system is a secure, web-based system that is available for free to institutional partners (www.projectredcap.org). Each clinical site will have a unique password and will enter data directly into the EDC system via a tablet supplied by the ASPiVLU trial.

The primary and secondary outcomes will be analysed according to intention-to-treat principles, i.e. by the treatment to which they were randomised. The primary outcome will be analysed with a log-rank test to compare the time to healing, up to the 12-week time point, i.e. with censoring at 12 weeks, between the two randomised treatment groups. A secondary analysis of the primary hypothesis will use logistic regression to compare between groups the proportion of participants healed by 12 weeks. The secondary outcome, the proportion of patients healed by 12 weeks and remaining recurrence-free at 24 weeks, will be compared between the two groups using logistic regression. The levels of inflammatory markers in the plasma will be compared between groups using a linear mixed model with a random effect for participants to allow for correlation among each individual’s repeat measures over time, adjustment for the biomarker’s level at randomisation, and an interaction between the time since randomisation and the randomisation group. The secondary outcomes of pain score and EQ-5D-5L will be analysed in linear mixed models, with a random effect for participant, an adjustment for the measure at randomisation, and an interaction between the time since randomisation and the randomisation group.

A secondary set of analyses will be performed to adjust for two predictors of healing: (1) ulcer area and ulcer duration at baseline and (2) any baseline characteristics that are found to be imbalanced between the groups to the extent of a 0.25 standard deviation difference in means (quantitative measures) or an odds ratio of 1.5 (binary measures). For time to healing these secondary analyses will use a Cox proportional hazards regression.

### Ethics approval

Full ethics approval has been granted from Alfred Health (HREC/14/Alfred/2 (Project 146/14)), Monash University (Project CF14/2106 - 2014001084), and Austin Health (HREC/14/Alfred/2 Project/15/Austin/55). Recruitment in any individual centre will not commence until local approvals are obtained. The ASPiVLU study will be conducted in accordance with the Declaration of Helsinki 1964, as revised in Edinburgh in 2008, the *Good Clinical Practice Guide* (CPMP/ICH/135/95) and with the National Health and Medical Research Council Guidelines on Human Experimentation.

## Discussion

This protocol outlines the design of a randomised, double-blinded, multicentre, placebo-controlled, clinical trial of aspirin in the management of VLUs. ASPiVLU will allow us to answer the question of whether daily active treatment with 300 mg aspirin improves time to healing of venous leg ulcers. The ASPiVLU sample will be sourced from four states in Australia, thereby providing a broad range of representation from the community. We will provide data on the efficacy and safety of aspirin in this population for a 24-week study period. Low-dose aspirin therapy may improve time to healing and decrease the number of recurrent VLU episodes. If proved effective, the low cost of aspirin therapy as an adjunct to compression would make it an affordable preventive agent for people with VLUs in all countries.

### Other ongoing aspirin studies

Two other randomised trials investigating aspirin in people with venous leg ulcers are ongoing: the AVURT (Aspirin for Venous Ulcers: Randomised Trial NCT02333123) and the Aspirin4VLU (Low-Dose Aspirin for Venous Leg Ulcers NTC 02158806). Chief investigators of the currently recruiting aspirin RCTs have formed the Aspirin for Venous Leg Ulcers Collaborative (AVLUC). AVLUC will combine data from the ASPiVLU study and the AVURT and Aspirin4VLU studies for individual patient data (IPD) meta-analysis.

### Current ASPiVLU study status

We have commenced recruitment at the Alfred, University of Tasmania, Melbourne Health, and Austin sites. Site-specific ethics approval from Prince Charles Hospital and Western Sydney Local Health are in progress, and all necessary ethical approvals for these participating sites will be obtained prior to study recruitment.

## Abbreviations

ABPI: ankle brachial pressure index
BMI: body mass index
CRF: case report form
DEPM: Department of Epidemiology and Preventive Medicine (Monash University)
DMC: data management committee
DSMB: data and safety monitoring board
CIDMU: Clinical Informatics and Data Management Unit
EDC: electronic data capture
VLU: venous leg ulcer
